# The other white‐nose syndrome transcriptome: Tolerant and susceptible hosts respond differently to the pathogen *Pseudogymnoascus destructans*

**DOI:** 10.1002/ece3.3234

**Published:** 2017-08-02

**Authors:** Christina M. Davy, Michael E. Donaldson, Craig K. R. Willis, Barry J. Saville, Liam P. McGuire, Heather Mayberry, Alana Wilcox, Gudrun Wibbelt, Vikram Misra, Trent Bollinger, Christopher J. Kyle

**Affiliations:** ^1^ Environmental and Life Sciences Graduate Program Trent University Peterborough ON Canada; ^2^ Department of Biology University of Winnipeg Winnipeg MB Canada; ^3^ Forensic Science Department Trent University Peterborough ON Canada; ^4^ Department of Biological Sciences Texas Tech University Lubbock TX USA; ^5^ Leibniz Institute of Zoo and Wildlife Research Berlin Germany; ^6^ Department of Microbiology Western College of Veterinary Medicine University of Saskatchewan Saskatoon SK Canada; ^7^ Department of Veterinary Pathology Western College of Veterinary Medicine University of Saskatchewan Saskatoon SK Canada; ^8^ Present address: Ontario Ministry of Natural Resources and Forestry, Wildlife Research and Monitoring Section Trent University Peterborough ON Canada

**Keywords:** coevolution, conservation genomics, emerging infectious diseases, gene expression, host–pathogen interactions, pathogenic fungi, susceptibility, tolerance resistance

## Abstract

Mitigation of emerging infectious diseases that threaten global biodiversity requires an understanding of critical host and pathogen responses to infection. For multihost pathogens where pathogen virulence or host susceptibility is variable, host–pathogen interactions in tolerant species may identify potential avenues for adaptive evolution in recently exposed, susceptible hosts. For example, the fungus *Pseudogymnoascus destructans* causes white‐nose syndrome (WNS) in hibernating bats and is responsible for catastrophic declines in some species in North America, where it was recently introduced. Bats in Europe and Asia, where the pathogen is endemic, are only mildly affected. Different environmental conditions among Nearctic and Palearctic hibernacula have been proposed as an explanation for variable disease outcomes, but this hypothesis has not been experimentally tested. We report the first controlled, experimental investigation of response to *P. destructans* in a tolerant, European species of bat (the greater mouse‐eared bat, *Myotis myotis*). We compared body condition, disease outcomes and gene expression in control (sham‐exposed) and exposed *M. myotis* that hibernated under controlled environmental conditions following treatment. Tolerant *M. myotis* experienced extremely limited fungal growth and did not exhibit symptoms of WNS. However, we detected no differential expression of genes associated with immune response in exposed bats, indicating that immune response does not drive tolerance of *P. destructans* in late hibernation. Variable responses to *P. destructans* among bat species cannot be attributed solely to environmental or ecological factors. Instead, our results implicate coevolution with the pathogen, and highlight the dynamic nature of the “white‐nose syndrome transcriptome.” Interspecific variation in response to exposure by the host (and possibly pathogen) emphasizes the importance of context in studies of the bat‐WNS system, and robust characterization of genetic responses to exposure in various hosts and the pathogen should precede any attempts to use particular bat species as generalizable “model hosts.”

## 1 INTRODUCTION

The impacts of pathogenic fungi on vertebrate hosts range widely, from mild symptoms in some circumstances, to rapid extinction in others (Ellison et al., 2015; Fisher et al., 2012; Hoyt et al., 2015; Langwig et al., 2015; Perez‐Nadales et al., 2014). Few interactions between fungal pathogens and vertebrate hosts are well understood, and these interactions are most often studied in susceptible species (Chen et al., 2013; Field et al., 2015). Generalizing results from these approaches could limit or misdirect the development of treatments, because pathogen virulence depends on complex interactions between the pathogen, the host, and the environment (James et al., 2015; Perez‐Nadales et al., 2014). Characterizing the response of tolerant or resistant vertebrate hosts to fungal infections can identify adaptive genes or processes linked to reduced disease severity or occurrence (Ellison et al., 2015; Rosenblum et al., 2012). These can then inform studies of susceptible species, including species recently exposed to the pathogen, where insufficient time has elapsed for selection for tolerance to occur.

There are two pathways by which tolerant, resistant, or susceptible hosts can differ in their molecular response to a pathogen. In the first scenario, all hosts upregulate the same biological response to a pathogen, but tolerant or resistant hosts possess particular alleles at a relevant gene that allow them to tolerate or prevent infection. A resistant and susceptible pair of frog species exposed to the fungal pathogen *Batrachochytridium dendrobatidis* upregulated the same biological processes, but experienced different disease outcomes (Rosenblum et al., 2012). Similarly, survival following exposure to *B. dentrobaditis* is correlated with particular MHC allele sequences in red‐eyed tree frogs (*Agalychnis callidryas*; Savage & Zamudio, 2011), implying that these alleles may confer tolerance to the pathogen. In the second scenario, tolerant/resistant and susceptible hosts upregulate different biological responses to infection. This scenario may account for different susceptibility to *B. dendrobatidis* in two toad species, in which resistant, infected toads upregulated genes related to skin integrity, but the susceptible, infected toads did not (Poorten & Rosenblum, 2016).

The pathogenic fungus *Pseudogymnoascus destructans* causes white‐nose syndrome (WNS) in hibernating bats and provides an excellent system for studying context‐dependent host–pathogen interactions (Brown, Schmid‐Hempel, & Schmid‐Hempel, 2003; Daskin & Alford, 2012). Pathogen virulence varies widely among host (bat) species, and the pathogen and its hosts trade advantageous conditions seasonally (Langwig et al., 2015). Bats are infected during hibernation when body temperature falls within the optimal range for growth of *P. destructans*. When bats emerge from hibernation, their body temperature rises rapidly to temperatures that inhibit growth of the pathogen (Verant et al., 2012). Recent introduction of *P. destructans* to North America has caused catastrophic population declines in some species. For example, the previously abundant little brown myotis (*Myotis lucifugus* LeConte 1831) was driven to “endangered” status in Canada in <10 years (Frick et al., 2015; Willis, 2015) and has declined precipitously in the northeastern United States (Langwig et al., 2012).

In contrast to the North American situation, European and Asian bats exhibit mild symptoms or remain asymptomatic following exposure to *P. destructans*, and no WNS‐related mass mortality has been documented on either continent (Hoyt et al., 2016a,b; Wibbelt et al., 2010; Zukal et al., 2016). These outcomes may reflect tolerance (the host experiences pathogen loads comparable to those of susceptible species, but does not exhibit severe disease symptoms), or resistance (the host exhibits significantly lower pathogen loads compared to susceptible species). Eurasian bats are considered tolerant to *P. destructans* (Zukal et al., 2016), presumably through coevolution with the pathogen (Leopardi, Blake, & Puechmaille, 2015).

The response of susceptible species to WNS has received substantial research attention. White‐nose syndrome causes mortality in susceptible bats by a disruption of hibernation behavior and physiological processes. These include increased arousal from torpor, hypotonic dehydration, and electrolyte imbalance (Reeder et al., 2012; Warnecke et al., 2012, 2013). Susceptible, North American *M. lucifugus* infected with *P. destructans* upregulate immune and inflammatory responses including cytokine and Toll‐like receptor activity, T‐cell recruitment, responses to wounding, and neutrophil aggregation (Field et al., 2015; Moore et al., 2013; Rapin et al., 2014). Bats that survive to emergence may exhibit immune response inflammatory syndrome as they mount a response to the pathogen (Meteyer, Barber, & Mandl, 2012). Carry‐over effects of WNS in susceptible bats include persistent increases in chronic stress in recovered *M. lucifugus* (Davy et al., 2016) and a potential decrease in reproductive success (Francl et al., 2012).

In tolerant species, tolerance has been primarily attributed to the varying environmental conditions in which different species hibernate (Hayman et al., 2016; Johnson et al., 2014), and to “coevolution with the pathogen” (Leopardi et al., 2015; Wibbelt et al., 2010). Persistent selective pressure by *P. destructans* (i.e., coevolution) can explain WNS tolerance in contemporary populations, but the molecular responses of species that have evolved tolerance of *P. destructans* have not been characterized. The mechanisms driving disease outcomes in susceptible and tolerant bat species exposed to *P. destructans* represent a critical gap in our understanding of this devastating pathogen (Cryan et al., 2013).

Here, we conducted an experimental exposure of a tolerant, European species of bat with *P. destructans*. We hibernated greater mouse‐eared bats (*M. myotis*) under controlled environmental conditions that are associated with severe disease outcomes following experimental exposure in a related, susceptible species (*M. lucifugus;* Warnecke et al., 2012). Controlling hibernation conditions allowed us to test the competing hypotheses that (1) tolerance of *P. destructans* is related to environmental conditions during hibernation (Hayman et al., 2016), predicting that “tolerant” bats should exhibit comparable disease outcomes when hibernating under the same conditions as susceptible species, or (2) tolerance to *P. destructans* is an inherent trait of some species, predicting that tolerant species will not develop severe disease even if exposed to high pathogen loads under controlled environmental conditions. We also hypothesized that susceptibility to *P. destructans* is determined by species‐specific interactions between the host and pathogen. This hypothesis predicts that the “white‐nose syndrome transcriptome” differs among hosts: That a tolerant European species of bat upregulate different biological processes following exposure to *P. destructans,* compared to a susceptible species. Finally, we attempted to apply a dual RNA‐seq approach to explore the response of *P. destructans* to a tolerant host.

## 2 MATERIALS AND METHODS

### 2.1 Sample collection

Collection and captive husbandry of live bats were carried out following animal care protocols approved by animal care committees at the Leibniz Institute of Zoo and Wildlife Research, the University of Saskatchewan, and the University of Winnipeg. All protocols complied with existing guidelines from the Canadian Council on Animal Care.

We collected juvenile, male greater mouse‐eared bats (*M. myotis* Borkhausen 1797) from colonies in Thuringia and Bavaria, Germany. This European species is tolerant to *P. destructans:* It only rarely develops symptoms of WNS, and no mass mortality from WNS is known in this species (Puechmaille et al., 2011; Wibbelt et al., 2010). Bats were held at the Federal Institute for Risk Assessment in Berlin, Germany, until transport to the Western College of Veterinary Medicine, University of Saskatchewan, Saskatoon, Canada, in 2012. We experimentally exposed *M. myotis* to *P. destructans* using the methods of Warnecke et al. (2012). Briefly, bats were randomly assigned to two groups. The wings of the first group (Mymy‐Neg; *n* = 8) were treated with a “sham” control treatment of PBS‐Tween 20 solution (Mymy‐Pos; *n* = 8). The wings of the second group were treated with fungal inoculum prepared from *P. destructans* collected in Atlantic Canada (500,000 conidia per μl in PBS‐Tween 20). Both groups then overwintered in captivity for 77 days under controlled conditions (7°C, >97% relative humidity) that replicated the conditions in which WNS develops in hibernating *M. lucifugus* (Supporting information). At the end of hibernation, the bats were euthanized humanely. Bats were weighed before and after hibernation to quantify effects of exposure to *P. destructans* on proportional weight loss. We swabbed the wings using sterile cotton swabs, and sampled whole‐wing tissue with surgical scissors following euthanasia. Wing tissue was placed directly in RNAlater, and stored at −80°C until RNA extraction. Swabs were tested for *P. destructans* using real‐time PCR (qPCR; Langwig et al., 2015; Muller et al., 2013). Histopathological symptoms of white‐nose syndrome (fungal colonization of the wings and epidermal cupping erosions) were investigated following methods described in Cheng et al. (2016).

The Supporting information details an independent experiment in which we applied these methods to *M. lucifugus,* confirming that our experimental treatment causes pathogen growth and clinical disease in a susceptible host. Briefly, *M. lucifugus* were collected from a *P. destructans*‐naïve hibernaculum in central Manitoba, Canada, in November 2013. We applied the same exposure methods as above to establish two uninfected and two *P. destructans*‐exposed treatment replicates (Mylu‐Neg and Mylu‐Pos; *n* = 10 in each group). The fungal inoculum was prepared from fresh *P. destructans* samples from Atlantic Canada. Bats hibernated in captivity (7°C, >97% relative humidity). At the endpoint of the experiment, fungal growth on exposed *M. lucifugus* was confirmed by ultraviolet fluorescence. Wing tissue was sampled from torpid bats at the endpoint of the experiment with 5‐mm biopsy punches, placed directly in RNAlater, and stored at ‐80 until RNA analysis (for details, see Supporting information).

### 2.2 RNA extraction

Total RNA was extracted from individual, whole‐wing *M. myotis* samples. All tissue samples were re‐suspended in TRIzol reagent (Invitrogen) and transferred to 2‐ml screw‐cap tubes containing Lysing Matrix D (MP Biomedicals). Tissue was disrupted using a FastPrep^®^‐24 Instrument (MP Biomedicals, speed setting = 6.5 for 45 s). Tissue homogenization was conducted three times, and tubes were cooled on ice for one min between cycles. Cell debris was pelleted by centrifugation at 12,200 × *g* for 10 min at 4°C, and the supernatant was transferred to 1.5 ml RNase‐free microcentrifuge tubes. Total RNA was isolated using TRIzol reagent according to the manufacturer's protocol. Total RNA was precipitated using RNA precipitation solution (0.8 mol/L disodium citrate/1.2 mol/L NaCl) and isopropanol (Sambrook & Russell, 2001), washed with 75% ethanol, and re‐suspended in nuclease‐free water (not DEPC‐treated; Ambion). Total RNA was treated with DNAseI (RNase‐free, New England Biolabs) and precipitated as above. We assessed the quality of *M. myotis* DNaseI‐treated total RNA following glyoxal denaturation using agarose gel electrophoresis (Sambrook & Russell, 2001).

### 2.3 cDNA library preparation and RNA‐sequencing

Total RNA was sent to The Centre for Applied Genomics at The Hospital for Sick Children (Toronto, Canada). RNA quality was assessed using a Bioanalyzer (Agilent Technologies). RNA Integrity Numbers (RIN) > 6.8 and DV200 (percentage of RNA fragments > 200 nt in size) values > 93% were confirmed for all samples. Poly(A) mRNA was enriched using oligo dT‐beads, and cDNA libraries were prepared using the TruSeq Stranded mRNA Library Preparation kit (Illumina Inc.). Libraries were checked on a Bioanalyzer for size, and to confirm primer dimers were rare or absent. Library quantification was carried out using the Library Quantification Kit—Illumina/ABI Prism (KAPA Biosystems) on a StepOne Plus real‐Time PCR System (Life Technologies). Based on these results, barcoded libraries were pooled in equimolar quantities and sequenced on a HiSeq 2500 System (Illumina Inc.) to generate 150 bp paired‐end reads. Base calling was performed using Casava Software v1.8.2 (Illumina Inc.). Sixteen *M. myotis* libraries were run on three lanes of Illumina sequencing.

### 2.4 RNA‐sequencing alignment and analysis

We assessed fastq sequence data quality using FastQC v0.11.5 (Andrews, 2010) and trimmed the reads to remove adapter sequences and low‐quality bases using Trimmomatic v0.36 (Bolger, Lohse, & Usadel, 2014) with the following settings: Illumina clop:2:30:10, leading:3, tailing:3, slidingwindow:4:15, minlength:36. The resulting trimmed paired‐end reads were combined prior to alignment. We originally planned to align reads to the available *M. lucifugus* genome (Myoluc2.0; http://www.ensembl.org/Myotis_lucifugus/Info/Index?db=core; Cunningham et al., 2015), but alignment of reads was low (~28%). We therefore used Trinity v2.2.0 (Grabherr et al., 2011) to generate a de novo transcript assembly in strand‐specific mode (RF) with silico read normalization. The TrinityStats perl script was used to generate transcript assembly statistics.

We used the Trinity “align_and_estimate_abundance” perl script to estimate expression levels for each transcript contig. This pipeline used Bowtie v1.1.2 (Langmead et al., 2009) to map the paired‐end reads from each sample to the de novo transcript assembly and RSEM v1.2.31 (Li & Dewey, 2011) to estimate the abundance of each transcript contig. We used the Trinity contig_ExN50_statistic perl script to calculate the ExN50 statistic. We identified differentially expressed transcripts between the control and exposed sample groups using SARTools v.1.3.0 (Varet, Coppée, & Dillies, 2016), which streamlined the DESeq2 v.1.12.3 (Love, Huber, & Anders, 2014) and edgeR v3.14.0 (Robinson, McCarthy, & Smyth, 2010) analyses. The SARTools‐based DESeq2 settings included cooksCutoff = TRUE (perform outliers detection), independentFiltering = TRUE, alpha = 0.05 (threshold of statistical significance), pAdjustMethod = BH (benjamini hochberg *p*‐value adjustment method; Benjamini and Hochberg, 1995), and locfunc = median (estimate size factors). The SARTools‐based edgeR settings included alpha = 0.05, pAdjustMethod = BH, cpmCutoff = 1 (counts‐per‐million cutoff), normalizationMethod = TMM (trimmed mean of *M*‐values used for normalization). Following the DESeq2 and edgeR analyses, we filtered the significant (adjusted *p* < .05) results to include only the differentially expressed transcript contigs with fold change >2. We performed hierarchical clustering of samples and transcript contigs using the Trinity PtR perl script, which utilized the hclust function with the complete‐linkage method. We conducted principal component analysis using SARTools, which transformed the RSEM‐estimated transcript contig counts using the variance stabilizing transformed (VST) method. Heatmaps were produced using the heatmap.2 function in the gplots v.3.5.0 package, using Pearson correlation as a similarity metric.

To characterize the transcriptome of *P. destructans* growing on a tolerant species, we used TopHat v2.1.1 (Kim et al., 2013) to align trimmed fastq files to the annotated Ensembl *M. lucifugus* genome sequence assembly (Myoluc2.0; Cunningham et al., 2015). Approximately 28% of the reads from each library aligned to the *M. lucifugus* genome. Next, we used BEDtools v2.17.0 (Quinlan & Hall, 2010) to extract reads that did not map to the *M. lucifugus* genome and aligned them using TopHat v2.1.1 to the annotated *P. destructans* genome sequence assembly (20631‐21; Broad Institute of Harvard and MIT, 2013).

### 2.5 Transcript contig annotation and gene ontology enrichment analysis

We conducted BLASTx sequence similarity searches of the NCBI nonredundant protein database (downloaded Oct 21, 2016), Swissprot protein database (downloaded Oct 21, 2016) and the Ensembl human protein database (downloaded Oct 26, 2016) using NCBI BLAST 2.5.0 +  (McGinnis & Madden, 2004) with an e‐value cutoff of 1E‐03 for transcript contigs identified as being differentially expressed (>2 fold change and FDR < 0.05) via the DESeq2 or edgeR analyses. We used the Ensembl human protein IDs identified in the DESeq2 or edgeR analyses as input for the web‐based g:Profiler (Reimand et al., 2016) to test for gene ontology (GO) term enrichment (Conesa et al., 2005), using a g:SCS significance threshold <0.05.

## 3 RESULTS

Tolerant *M. myotis* exposed to *P. destructans* and hibernated under controlled environmental conditions exhibited no obvious symptoms of WNS. Body weight in the two treatment groups was similar prior to exposure, and remained similar at the endpoint (pretreatment: *t* = −1.076, *df* = 14, two‐tailed *p* = .300; endpoint: *t* = 0.843; *df *= 14; two‐tailed *p* = .419). Bats that were exposed to *P. destructans* retained a greater proportion of body weight during hibernation (*t* = 2.630; *df* = 14, two‐tailed *p* = .019). No fungal growth was superficially visible on the wings of exposed bats at the endpoint of the experiment, and qPCR detected *P. destructans* on the wings of only three bats (MymyPos3, 4 and 5; ct values ranged from 33.064 to 40.068). Histopathology identified extremely limited fungal growth on the wings, and no conidia or cupping erosions of the wing tissue were observed. In contrast, the application of our methods to susceptible *M. lucifugus* hibernating under the same environmental conditions resulted in high fungal loads from which *P. destructans* RNA could be isolated and sequenced, and caused clinical WNS characterized by lethargic behavior and substantial, obvious fungal lesions on the wings that fluoresced under UV light (Warnecke et al., 2012; Supporting information).

**Figure 1:**
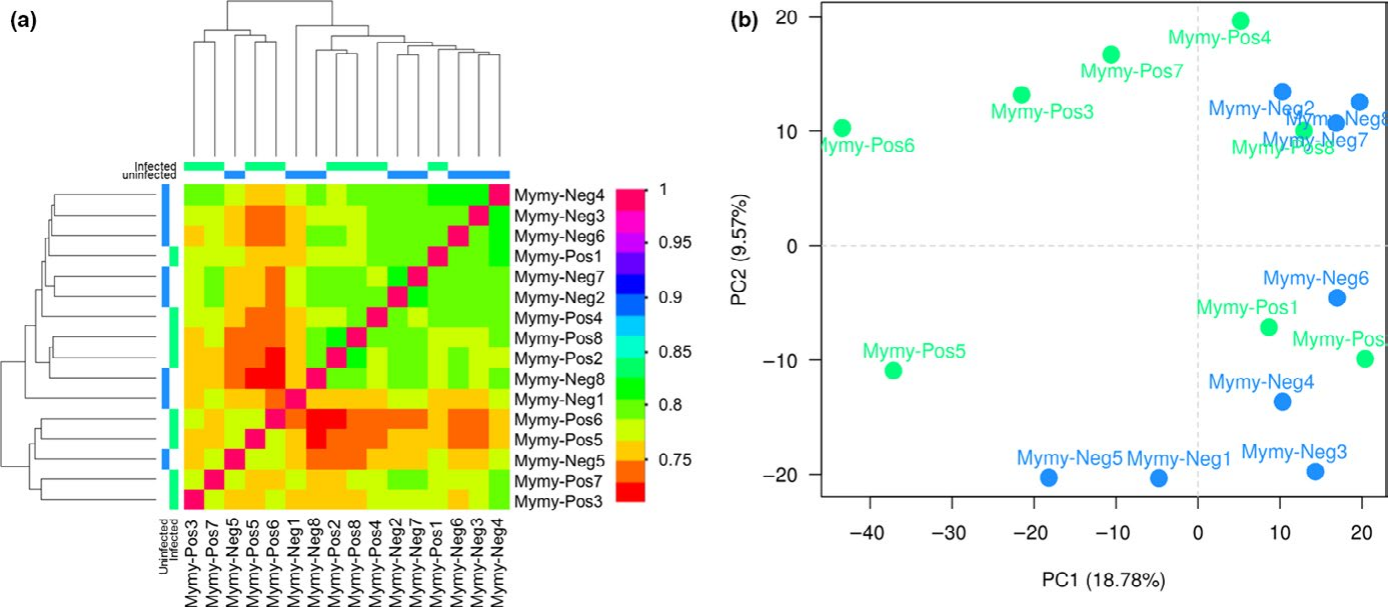
Variation in gene expression between *Myotis myotis* that are unexposed (Mymy‐Neg) or experimentally exposed (Mymy‐Pos) to *Pseudogymnoascus destructans*. (a) Hierarchical clustering of RSEM‐estimated transcript contig counts using Pearson correlation complete‐linkage clustering. Colored bars above and to the left of the heatmap indicate control (blue) or exposed (green) samples. Scale shows Pearson correlation coefficient. (b) Principal component analysis on variance stabilizing transformed RSEM‐estimated transcript contig counts. Percentages of variance associated with each axis are provided. Blue spheres represent control bats and green spheres represent exposed bats

**Figure 2:**
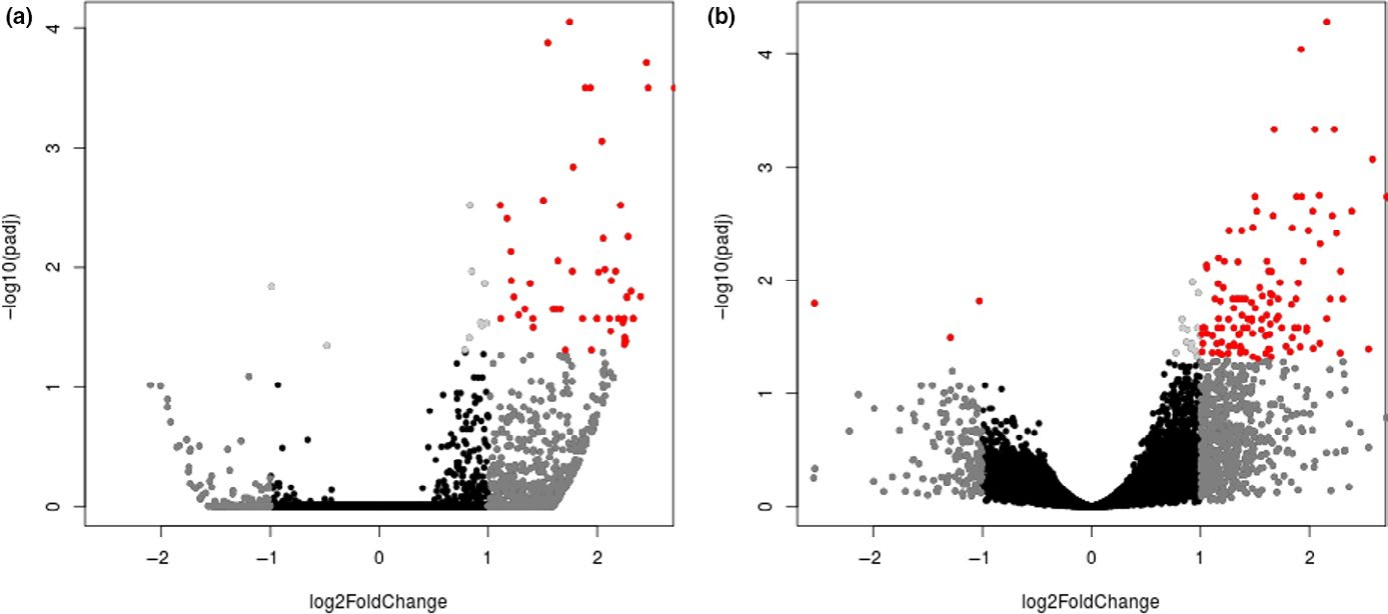
Differential expression between control (Mymy‐Pos) and exposed (Mymy‐Neg) treatments illustrated with volcano plots, showing the log of the adjusted *p*‐value as a function of the log ratio of differential expression based on (a) RSEM and DESeq2, and (b) RSEM and edgeR. Colored data points plot groups of genes based on fold change and FDR cutoff: red (>2 fold change, FDR < 0.05), dark gray (>2 fold change, FDR > 0.05), light gray (<2 fold change, FDR < 0.05), black (<2 fold change, FDR > 0.05)

**Figure 3:**
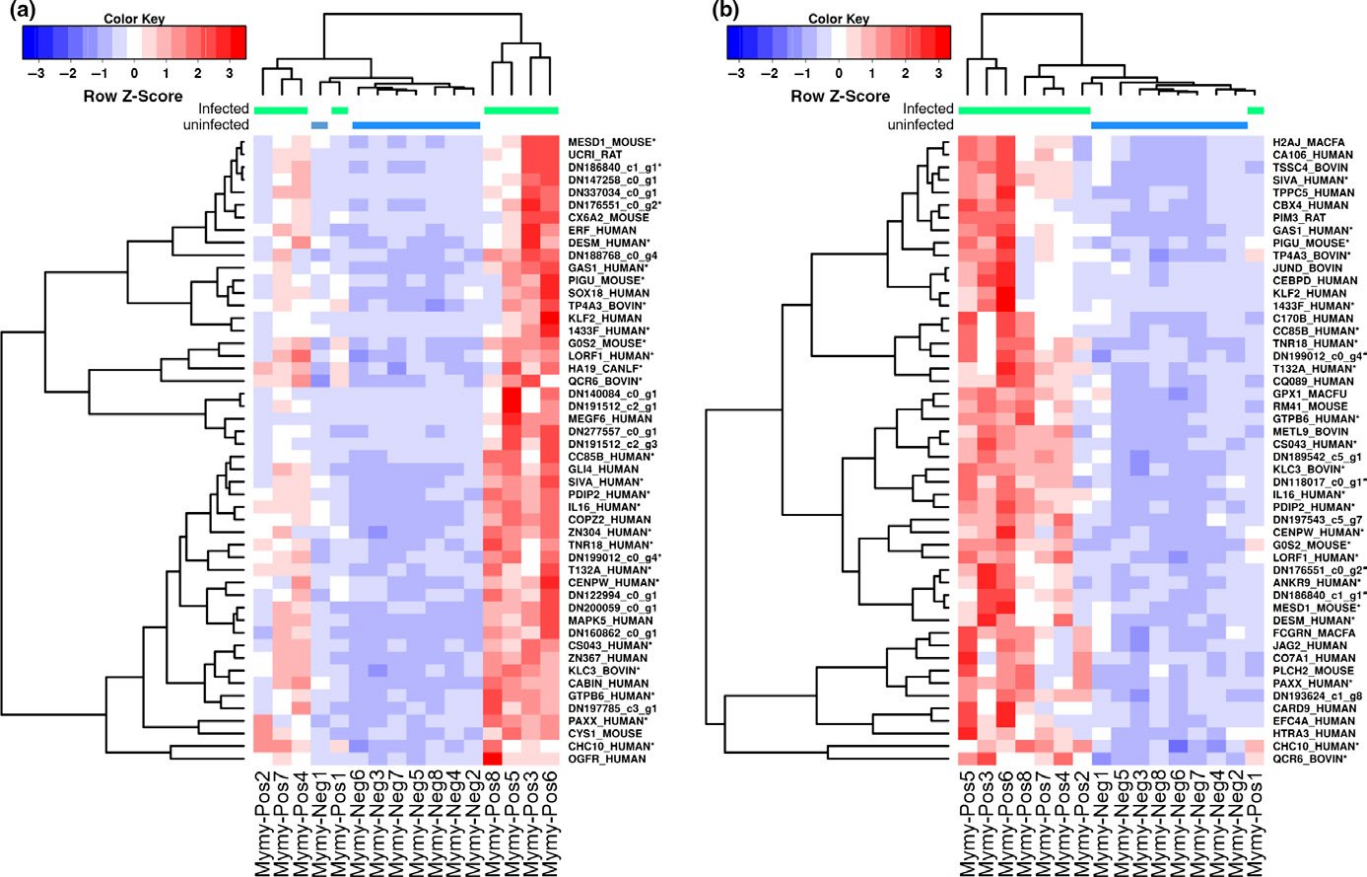
Transcriptional analysis of *Myotis myotis* unexposed or experimentally exposed to *Pseudogymnoascus destructans* (Mymy‐Neg; Mymy‐Pos). Centered *Z*‐scores of TMM‐normalized RSEM‐estimated gene counts for the 50 most significant differentially expressed genes identified by (a) RSEM and DESeq2 and (b) RSEM and edgeR. Adjusted *p*‐values ranged from 2.68E−02 to 8.84E−05 and 1.46E−02 to 5.21E−05 for the analyses conducted in (a) and (b), respectively. Hierarchical clustering of differentially expressed genes and samples used Pearson correlation as a similarity metric. Colored bars above the heatmap indicate control (unexposed; blue) or exposed (green) samples. Where possible, transcripts were identified by blastx alignment to the SwissProt database, and Trinity‐based transcript contig identifiers are used elsewhere

We generated 16 strand‐specific libraries, and Illumina sequencing of these produced ~459 million raw paired‐end reads. Removal of low‐quality bases and contaminating adapter sequences left ~341 million trimmed paired‐end reads that were used for further analysis (Table S1). The de novo *M. myotis* transcriptome assembly contained 757,963 genes (980,944 transcripts). Overall, 50% of the assembled bases were found in transcript contigs at least 1,502 bases in length (N50; Appendix S1). Further, when the N50 statistic was limited to the most highly expressed transcripts, the maximum contig length was calculated to be 3,018 bases in length, representing 79% of the total normalized expression data (or 34,883 transcripts; Table S2). Average alignment of trimmed paired‐end reads from each library to the de novo *M. myotis* transcriptome assembly was 76.6% (Table S1). The correlation matrix generated by RSEM did not resolve the samples based on treatment groups using hierarchical clustering (Fig. 1a). In the PCA the first two principal components only accounted for ~28% of variation among the samples (Fig. 1b), and also did not cluster the samples based on treatment. The three exposed bats (MymyPos3, ‐4, and ‐5) on which qPCR detected *P. destructans* did not cluster together in based on the RNA‐seq data (Figs 1, 2, 3).

We aligned the remaining trimmed, paired‐end reads to the *P. destructans* genome assembly, but *P. destructans* was almost undetectable in the exposed *M. myotis* libraries. Specifically, only 147 trimmed paired‐end reads mapped to the *P. destructans* genome, consistent with the limited fungal growth observed. We were therefore unable to characterize the *P. destructans* transcriptome during *M. myotis* infection.

Differential gene expression (Fig. 2) between control and exposed *M. myotis* samples is summarized in Table S3 (DESeq2 analysis) and Table S4 (edgeR analysis). Using DESeq2, we found 59 transcript clusters expressed at higher levels in the exposed bat tissue; no transcript clusters were expressed at lower levels. Using edgeR, we found 128 transcript clusters expressed at higher levels in the exposed bat tissue and four transcript clusters that were expressed at lower levels (Table S3). Only 28 transcript clusters overlapped between the two analyses. When combined, the two analyses contained a total of 163 differentially expressed genes, 128 of which had significant sequence similarity (*e*‐value < 1E−03) to characterized proteins in the Swissprot database or to proteins in the human genome assembly (GRCh38; Table S5). We used BLASTx to annotate ten additional transcript contigs with significant sequence similarity to the nonredundant NCBI database (data not shown); however, the remaining 25 transcript clusters could not be identified based on sequence similarity to known proteins. Figure 3 shows TMM‐normalized counts for transcript clusters with the 50 lowest adjusted p‐values based on the DESeq2 (Fig. 3a) and edgeR (Fig. 3b) analyses. In the edgeR analysis, hierarchical clustering separated most of the samples based on treatment group, except for Mymy‐Pos1, which was grouped with the Mymy‐Neg samples. Tests for GO‐term enrichment identified no enriched processes using the DESeq2‐derived gene set. Analysis based on the edgeR‐derived gene set identified only two enriched processes (movement of cell or subcellular component and negative regulation of cellular process; Table S6), neither of which is of obvious biological significance in the host response to *P. destructans*.

## 4 DISCUSSION

Exposure to high doses of *P. destructans* did not cause *M. myotis* to develop symptoms of WNS, despite hibernating under controlled environmental conditions that mimicked those selected by wild, susceptible *M. lucifugus*. Although *P. destructans* exhibits rapid growth and causes disease in susceptible species under the same environmental conditions, the fungus exhibited extremely low activity on tolerant bats, becoming virtually undetectable after 77 days of hibernation. Microhabitat selection during hibernation may explain variable outcomes in tolerant bats (Moore et al., 2013; Zukal et al., 2016), but our results do not support the hypothesis that tolerance to WNS is caused in part by host microhabitat selection (Hayman et al., 2016). Instead, our data suggest that tolerance is an inherent (i.e., genetic) trait of some species, implying that tolerance could potentially evolve in species that are currently susceptible. Our data also highlight the dynamic nature of the “WNS transcriptome,” which varies among host species and may also vary temporally throughout the process of infection and the development of disease. The remarkably low growth of *P. desctructans* precluded us from characterizing its transcriptome during response to a tolerant host. The lack of significant activity by *P. destructans* growing on *M. myotis,* despite optimal temperature and humidity for its growth, suggests that the pathogen may respond differently to tolerant and susceptible hosts. Context‐dependent responses to infection by both the host (bats) and pathogen (*P. destructans*) provide an exciting direction for future research.

Gene expression by tolerant *M. myotis* in response to *P. destructans* differs from that described in susceptible, North American *M. lucifugus* (Field et al., 2015; Supporting information). We detected no immune response to infection in tolerant *M. myotis*; in fact, we detected no substantial response to the pathogen at all. In contrast, *M. lucifugus* upregulate an array of immune and other physiological responses following exposure to *P. destructans*. Although our experiment cannot rule out a strong initial immune response to exposure in tolerant species, we detected no evidence of meaningful immune response to the fungus by the end of hibernation. The remarkable disparity in the response of *M. myotis* and *M. lucifugus* (this study; Field et al., 2015) illustrates the dynamic nature of the white‐nose syndrome transcriptome—especially the importance of context (e.g., host identity) to the response of the host, and potentially the pathogen.

While we cannot rule out potential variation in viability or virulence of the fungal spores between the *M. myotis* and *M. lucifugus* experiments, we are confident that the spores used were viable because spores harvested concurrently from the same fungal culture and stored under the same conditions were successfully grown in a subsequent, unrelated experiment conducted by V. Misra. Thus, extremely low detection of *P. destructans* on exposed, tolerant hosts, and the absence of conidia or cupping erosions detected during histopathological examination implies potential inhibition of pathogen activity. Tolerant hosts may not need to mount an immune response if they are able to repress *P. destructans* growth and pathogenicity, which could potentially be accomplished through secreted skin proteins or through the secretions of commensal bacteria (Hoyt et al., 2015, 2016a,b). We note that our results suggest potential mechanisms of resistance as well as tolerance, because we did not observe pathogen loads on *M. myotis* comparable to those seen on *M. lucifugus* exposed following the same protocol (Supporting information).

Wild *M. myotis* do often develop mild symptoms of WNS, although disease prevalence may vary among regions (Hoyt et al., 2016a,b; Wibbelt et al., 2010; Zukal et al., 2016). It is not yet clear what drives this variation. The main finding of our study is that controlling environmental conditions during hibernation does not reduce the tolerance or resistance of *M. myotis* to *P. destructans*, and that the difference in susceptibility of North American species such as *M. lucifugus* therefore cannot be attributed solely to differing microhabitat selection among species. However, this result does not exclude the potential influence of environmental conditions on the development of clinical symptoms among wild populations of tolerant species.

Our results are broadly compatible with the hypothesis of strong, pathogen‐mediated selective pressure on Eurasian bat species evolving in sympatry with *P. destructans*. However, our results do not implicate a role for “tolerance alleles” in conferring protection against WNS. Instead, we observed completely different biological processes (or lack thereof) in response to the pathogen, compared to a susceptible species. We hypothesize that differences in the regulatory regions of key immune genes among species may be involved in these different responses, but further research would be required to test this hypothesis. We note that our experiment, and others to date, capture only a single part of the complex host–pathogen interactions involved in white‐nose syndrome. Data from a susceptible and tolerant species at several intervals postinfection are required to understand the genetic basis for disease progression or suppression, and to test the assumption that *M. myotis* and *M. lucifugus* can serve as model “tolerant” or “susceptible” species in this system. Repeating our analyses with samples from tolerant hosts that are exhibiting clinical infection will reveal the range of responses that these species use to survive WNS. Comparison of samples taken from a variety of species at a late stage of infection could be particularly informative, because they could reveal the molecular mechanisms of infection in more susceptible individuals from tolerant species. Finally, most experimental exposures to date have used males in an effort to minimize impact on the demographic rates of wild populations (Warnecke et al., 2012; Field et al., 2015; this study). We encourage this approach—but the assumption that males and females mount similar responses to WNS should be explicitly tested.

We encourage future research to target the initial responses of host and pathogen to exposure—a critical time point that has yet to be been addressed in any study of WNS. The mechanisms that inhibit or promote pathogenesis in the fungus are most likely to be upregulated immediately following contact between host and pathogen. Investigating early‐stage interactions between bats and *P. destructans* could also disentangle the early responses of susceptible hosts (which are insufficient to prevent disease) from the host's response to severe disease and the associated physiological effects of infection. Interspecific variation in the initial response of bats to *P. destructans* could be directly tested by sampling resistant and susceptible bats shortly after exposure, prior to the development of clinical WNS. Such comparisons would ideally include North American species such as *Eptesicus fuscus* or *Corynorhinos townsendii virginianus* that may be more tolerant or resistant to infection with *P. destructans* (Frank et al., 2014; Hoyt et al., 2016a,b).

Finally, if recently developed methods for transcriptome characterization from small samples of whole blood (Huang et al., 2015) can be applied to infected wing biopsies, this will allow individual‐level analysis of bat's response to WNS and extended time series sampling, accounting more accurately for individual variations in response to infection. Time series sampling can also address another likely driver of host–pathogen interactions. In the bat‐WNS system, we may expect context‐dependent responses to dramatic shifts in the host's physiological state. Bats are susceptible to *P. destructans* while hibernating because their body temperature drops to the optimal temperature range for the fungus to grow. However, when bats emerge and increase their temperature the fungus is suddenly growing in a hostile environment. A preliminary transcriptomic analysis of *P. destructans* growing on *M. lucifugus* immediately before emergence and 48 hr after emergence revealed substantial shifts in pathogen response based on the physiological state of the host (Supporting information). These preliminary data once again highlight the fluid nature of host–pathogen interactions, and the importance of considering context when interpreting transcriptomic data.

Time is of the essence as the research community develops mitigations for WNS and other devastating epizootics (Jones et al., [Link]; Langwig et al., 2015), including treatments, vaccines, and measures to slow the spread of pathogens. Comparative transcriptomics can disentangle environmental effects on disease outcomes (e.g., Hoyt et al., 2016a,b; Langwig et al., 2012) from effects mediated by context‐specific host and pathogen responses to infection (Brown et al., 2003; Poorten & Rosenblum, 2016). Appreciating the importance of context and evolutionary history in host–pathogen interactions is critical to our ability to mitigate impacts of emerging infectious diseases on biodiversity, human health, agricultural systems, and ecosystem services (Enguita et al., 2016; Jones et al., [Link]; Smith, Sax, & Lafferty, 2006).

## CONFLICT OF INTEREST

None declared.

## DATA ACCESSIBILITY

Transcripts identified as significant are listed in searchable.xlsx databases (Tables S1‐6 and S7‐14). All RNA‐seq data will be deposited at the Sequence Read Archive (SRA; *accession number will be provided after manuscript acceptance*).
