# Supplementary material for: The other white‐nose syndrome transcriptome: Tolerant and susceptible hosts respond differently to the pathogen Pseudogymnoascus destructans
Source: Ecol Evol. 2017 Aug 2;7(18):7161–70. doi: 10.1002/ece3.3234 (PMC5606880; doi:10.1002/ece3.3234)
Supplement: Supplementary file 3 [file ECE3-7-7161-s003.docx]

**S1 Appendix. Trinity transcriptome contig Nx statistics.**

################################

## Counts of transcripts, etc.

################################

Total trinity 'genes': 757963

Total trinity transcripts: 980944

Percent GC: 47.81

########################################

Stats based on ALL transcript contigs:

########################################

Contig N10: 5496

Contig N20: 3796

Contig N30: 2765

Contig N40: 2041

Contig N50: 1502

Median contig length: 454

Average contig: 872.68

Total assembled bases: 856045596

#####################################################

## Stats based on ONLY LONGEST ISOFORM per 'GENE':

#####################################################

Contig N10: 3973

Contig N20: 2300

Contig N30: 1514

Contig N40: 1073

Contig N50: 791

Median contig length: 382

Average contig: 619.44

Total assembled bases: 469513446
