# Supplementary material for: The other white‐nose syndrome transcriptome: Tolerant and susceptible hosts respond differently to the pathogen Pseudogymnoascus destructans
Source: Ecol Evol. 2017 Aug 2;7(18):7161–70. doi: 10.1002/ece3.3234 (PMC5606880; doi:10.1002/ece3.3234)
Supplement: Supplementary file 4 [file ECE3-7-7161-s004.docx]

**Supporting information for:**

**The Other White-Nose Syndrome Transcriptome: tolerant and susceptible hosts respond differently to infection with *Pseudogymnoascus destructans***

**Introduction**

Our initial goal was to pair the *Myotis myotis* study with an equally controlled experimental inoculation of susceptible *Myotis lucifugus.* We simultaneously attempted to address the response of infected bats and *P. destructans* to host emergence from hibernation. This required time-series sampling of bat wing tissue. Unfortunately, we were unable to isolate sufficient RNA for analysis from individual wing biopsies from *M. lucifugus.* Tests of RNA extraction efficiency showed that our methods required a minimum of 10 punches per extraction to provide sufficient RNA quantity for sequencing. Therefore, we pooled the 10 samples within each treatment for analysis. RNA-seq results from the *M. lucifugus* illustrate significant changes in host-pathogen interactions following exposure to *P. destructans* as well as characterizing gene expression by *P. destructans* on susceptible bats under controlled environmental conditions. However, we acknowledge that the required sample pooling affected our original experimental design, and that our *M. lucifugus* results that require further replication in a future study.

We therefore present the results of this concurrent experiment here as preliminary data, as a supplemental to the *M. myotis* study. The main outcome of the concurrent experiment is to confirm that our methods result in pathogen growth and clinical WNS on susceptible hosts- in other words, to confirm that different responses of *M. myotis* and *M. lucifugus* to pathogen exposure can be attributed to host identity, rather than experimental flaws.

In our preliminary experiment, we also investigated the effects of changes in host physiology (emergence from hibernation) on gene expression in susceptible bats (*Myotis lucifugus*) infected with *Pseudogymnoascus destructans.* Our goal was to test the hypothesis that the transcriptome of both the host and pathogen shifts following emergence. During torpor, the reduced body temperature of the host provides optimal conditions for pathogen growth. Following arousal from hibernation, *P. destructans* finds itself growing in a hostile environment as host body temperature rises to temperatures that inhibit the growth of this species.

**Methods**

*Sample collection*

Male little Brown Bats (*Myotis lucifugus*) were collected from a *P. destructans-*naïve hibernaculum in central Manitoba, Canada in November 2013. We replicated the inoculation methods described in the main paper (following Warnecke et al, 2012) to establish two uninfected and two *P. destructans-*inoculated treatment replicates (Mylu-Neg and Mylu-Pos; n = 10 in each group). The fungal inoculum was prepared from fresh *P. destructans* samples from Atlantic Canada, to avoid confounding effects of potential attenuated virulence that may result from long-term storage at –80°C. Bats hibernated in captivity (7°C, > 97% relative humidity) and wing tissue was sampled from torpid bats at the endpoint the experiment with 5 mm biopsy punches and placed directly in RNAlater.

To investigate how *P. destructans* responds to host emergence from hibernation, we re-sampled bats from both groups48 hours post-emergence (PE) when the bats had become normothermic (Mylu-Neg-PE, Mylu-Pos-PE). Samples were stored at -80°C until RNA extraction. Fungal growth on infected *M. lucifugus* was confirmed by ultraviolet fluorescence. Extraction of RNA from pooled samples, RNA sequencing, and gene expression analyses followed the methods described in the main document, with the exception that reads were aligned directly to the annotated *M. lucifugus* genome sequence assembly Myoluc2.0 (<http://www.ensembl.org/Myotis_lucifugus/Info/Index?db=core>; Cunningham et al., 2015) using gene models predicted by Ensembl. The percentage of mapped reads was 63% for *M. lucifugus.*

To distinguish fungal transcripts from host orthologs, we used BEDtools v2.17.0 (Quinlan & Hall, 2010) to extract reads that did not map to the *M. lucifugus* genome and then aligned them using TopHat to the annotated *P. destructans* genome sequence assembly v20631-21 (MIT, 2013), using gene models predicted by The Broad Institute of MIT and Harvard. To identify transcripts expressed by the pathogen in *P. destructans*-infected wing samples, we used Cufflinks v2.2.1 to estimate expression levels as FPKM (fragments per kilobase of transcript per million reads mapped) and Cuffnorm to transform those values using geometric normalization. We explored the *P. destructans* expression data using cutoffs of normalized FPKM (nFPKM) > 1, > 10, > 100, > 1000, and > 10000. *Pseudogymnoascus destructans* datasets were scanned for GO-term enrichment using Fungifun2 (Priebe, Kreisel, Horn, Guthke, & Linde, 2015) and a Fisher’s Exact Test with FDR<0.05.

We used portions of the Trinotate annotation suite (<http://trinotate.github.io/>) to further characterize *P. destructans* proteins beyond existing annotations, including: blastp sequence similarity search to known sequence data (SwissProt database, February 6, 2015) and protein signal peptide prediction (singalP v4.0) (Petersen, Brunak, von Heijne, & Nielsen, 2011). Local blastp searches were conducted against proteins from human pathogenic fungi, including: *Aspergillus fumigatus* (CADRE.26.pep.all.fasta), *Aspergillus nidulans* (ASM1142v1.26.pep.all.fasta), *Candida albicans* (SC5314_A22_current_orf_trans_all.fasta), and *Cryptococcus neoformans* (GCA_000091045.1.26.pep.all.fasta). All blastp searches were conducted with an e-value cutoff of 1^-3^.

**Results**

*Response of* M. lucifugus *to infection*

Inoculated *M. lucifugus* sampled at the end of hibernation did not exhibit significant proportional weight loss relative to control bats (t = 0.142, df = 18; p = 0.888), but did exhibit severe symptoms of WNS including lethargic behaviour, and substantial, obvious fungal lesions on the wings that fluoresced under UV light.

RNA extraction was successful for three samples but failed for the Mylu-Neg-PE sample. Illumina RNA-seq of strand-specific libraries generated > 65 million reads that aligned to the *M. lucifugus* genome (Table S7). Hierarchical clustering analysis separated the biological replicates (Fig. 3a, S2a). Depending on the analysis method, 126 (DESeq2; Fig. 3b, S3c) to 226 (edgeR; Fig. S2b, S3d) genes were differentially expressed between the uninfected and Pd-infected sample groups (>2-fold change, FDR <0.05). All 126 DESeq2-derived genes were in the edgeR-derived gene-set (Fig. 1b and Table S4). Enrichment of Gene Ontology (GO) terms linked to these transcripts revealed that infected *M. lucifugus* up-regulated biological processes including immune pathways (T cell activation, chemokines and cytokine receptors), defense response, inflammatory responses, and response to wounding (Table S8). Only three genes expressed in *M. myotis* in response to exposure were also expressed in infected *M. lucifugus* (SOCS3, IRF1, IER5L), indicating substantial inter-specific variation in response to *P. destructans*.

*Response of infected* M. lucifugus *to emergence from hibernation*

Based on a dispersion of 0.1, edgeR identified 998 differentially expressed transcripts between Mylu-Pos and Mylu-Pos-PE (Table S9), indicating a rapid, substantial shift in gene expression associated with a number of biological processes related to blood vessel and circulatory system development following emergence (Table S10). We note that this result should be considered anecdotal until it can be robustly replicated, but it provides a preliminary glimpse into shifts in gene expression by infected bats directly following emergence.

*Response of* P. destructans *to physiological shifts in a susceptible host*

Over 1.2 million reads aligned to the *P. destructans* genome from infected *M. lucifugus* sampled before and after emergence from hibernation (Table S11). We tested for GO-term enrichment in transcripts with a high normalized FPKM (nFPKM) values (Table S12), which included many that encode putative secreted proteins (Table S13 compares secreted protein detection between our data and previously published studies). Many of the detected *P. destructans* transcripts encode proteins similar to reference proteins in the SWISSPROT database; including several pathogenesis-related proteins such as *A. fumigatus* allergen Asp f2 and allergen Asp f7, and the *C. albicans* Pry family pathogenesis-related protein C1_07040C_B. To test for GO-term enrichment we conservatively used the 1074 transcripts that had a high nFPKM values and identified terms related to active growth (Table S10). Many previously characterized transcripts encoding putative secreted virulence factors were also detected including Destructin-1 and -2, but not Destructin-3 (Field et al., 2015; O’Donoghue et al., 2015; Pannkuk et al., 2015; Table S14).

*Response of P. destructans to physiological shifts in the host*

We manually set the edgeR and cufflinks dispersion settings as described above to identify putative differentially expressed *P. destructans* genes following host emergence from hibernation. We identified 113 differentially-expressed fungal transcripts between the pre- and post-emergence samples, including 31 genes which encode putative secreted proteins (Table S14). GO-term enrichment revealed several possible virulence-related GO terms, including hydrolase activity (Monod et al., 2002; Schaller, Borelli, Korting, & Hube, 2005), peptidase activity, and heme binding (Table S13).

**Preliminary Findings**

*Myotis lucifugus* exposed to *P. destructans* following our protocol developed visible fungal loads and clinical symptoms of WNS, confirming that the different response we observed in *M. myotis* can be attributed to host identity rather than confounding factors such as temperature or humidity. Gene expression in *P. destructans* shifted significantly in only 48 hours between the end of hibernation and the up-regulation of biological processes associated with host activity, including dramatic increases in body temperature. Our initial goal was to directly compare our results from *M. lucifugus* to those of our main study with *Myotis myotis,* because the controlled conditions under which the bat hibernated provided a unique opportunity to control potentially confounding environmental factors that have not been accounted for in previous studies. We were unfortunately forced to pool samples from *M. lucifugus,* because we were unable to isolate sufficient RNA from single biopsy punches. The resulting lack of replication is obviously problematic because samples from 10 individuals pooled and sequenced together represent an effective sample size of 1. Nevertheless, these results provide further illustration that the “white-nose syndrome transcriptome” is a complex, constantly shifting target. We include them here with the goal of inspiring further research into context-dependent host-pathogen interactions.

**Data Accessibility:** Transcripts identified as significant are listed in a searchable .xls database (Supplemental Tables S7-14). All RNA-seq data will be deposited at the Sequence Read Archive (SRA; accession number XXXXX)

**References**

Cunningham, F., Amode, M. R., Barrell, D., Beal, K., Billis, K., Brent, S., et al. (2015). Ensembl 2015. Nucleic Acids Res. 43, D662–D669. doi:10.1093/nar/gku1010.

Field, K. A., Johnson, J. S., Lilley, T. M., Reeder, S. M., Rogers, E. J., Behr, M. J., et al. (2015). The White-Nose Syndrome Transcriptome: Activation of Anti-fungal Host Responses in Wing Tissue of Hibernating Little Brown Myotis. PLoS Pathog 11, e1005168–e1005168. Available at: http://dx.doi.org/10.1371%2Fjournal.ppat.1005168.

MIT, B. I. of H. and (2013). Geomyces destructans Sequencing Project. Available at: http://www.broadinstitute.org.

Monod, M., Capoccia, S., Léchenne, B., Zaugg, C., Holdom, M., and Jousson, O. (2002). Secreted proteases from pathogenic fungi. Int. J. Med. Microbiol. 292, 405–419. doi:10.1078/1438-4221-00223.

O’Donoghue, A. J., Knudsen, G. M., Beekman, C., Perry, J. a., Johnson, A. D., DeRisi, J. L., et al. (2015). Destructin-1 is a collagen-degrading endopeptidase secreted by Pseudogymnoascus destructans, the causative agent of white-nose syndrome. Proc. Natl. Acad. Sci. 112, 7478–7483. doi:10.1073/pnas.1507082112.

Pannkuk, E. L., Risch, T. S., and Savary, B. J. (2015). Isolation and Identification of an Extracellular Subtilisin-Like Serine Protease Secreted by the Bat Pathogen <italic>Pseudogymnoascus destructans</italic>. PLoS One 10, e0120508. doi:10.1371/journal.pone.0120508.

Petersen, T. N., Brunak, S., von Heijne, G., and Nielsen, H. (2011). SignalP 4.0: discriminating signal peptides from transmembrane regions. Nat. Methods 8, 785–786. doi:10.1038/nmeth.1701; 10.1038/nmeth.1701.

Priebe, S., Kreisel, C., Horn, F., Guthke, R., and Linde, J. (2015). FungiFun2: a comprehensive online resource for systematic analysis of gene lists from fungal species. Bioinforma. 31 , 445–446. doi:10.1093/bioinformatics/btu627.

Quinlan, A. R., and Hall, I. M. (2010). BEDTools: a flexible suite of utilities for comparing genomic features. Bioinforma. 26 , 841–842. doi:10.1093/bioinformatics/btq033.

Schaller, M., Borelli, C., Korting, H. C., and Hube, B. (2005). Hydrolytic enzymes as virulence factors of Candida albicans. Mycoses 48, 365–377. doi:10.1111/j.1439-0507.2005.01165.x.

Warnecke, L., Turner, J.M., Bollinger, T.K., Lorch, J.M., Misra, V., Cryan, P.M., Wibbelt, G., Blehert, D.S. and Willis, C.K., 2012. Inoculation of bats with European Geomyces destructans supports the novel pathogen hypothesis for the origin of white-nose syndrome. Proceedings of the National Academy of Sciences, 109(18), pp.6999-7003.

atabase S11inding (Database S13
atabase S11inding (Database S13
atabase S11inding (Database S13
atabase S11inding (Database S13
atabase S11inding (Database S13
